# Oxidative Status as an Indicator of Gonadal Maturation in Three Species of Mediterranean Sea Urchin

**DOI:** 10.3390/antiox15040516

**Published:** 2026-04-21

**Authors:** Pedro A. Álvarez, Alberto Coll, María Elena Díaz-Casado, Félix Hidalgo, Eva E. Rufino-Palomares, Amalia Pérez-Jiménez, Cristina E. Trenzado

**Affiliations:** 1 Servicio de Desarrollo Pesquero, Delegación Territorial de Agricultura, Pesca, Agua y Desarrollo Rural en Granada, Junta de Andalucía, 18013 Granada, Spain; pedroa.alvarez@juntadeandalucia.es; 2 Departamento de Biología Celular, Universidad de Granada, 18071 Granada, Spain; albertocoll@ugr.es; 3 Departamento de Fisiología, Universidad de Granada, 18071 Granada, Spain; elenadiaz@ugr.es; 4 Departamento de Zoología, Universidad de Granada, 18071 Granada, Spain; fhidalgo@ugr.es (F.H.); calaya@ugr.es (A.P.-J.); 5 Departamento de Bioquímica y Biología Molecular I, Universidad de Granada, 18071 Granada, Spain; evaevae@ugr.es

**Keywords:** antioxidants, *Arbacia lixula*, digestive, gonads, metabolism, oxidative stress, *Paracentrotus lividus*, sea urchin, *Sphaerichinus granularis*

## Abstract

Sea urchins are invertebrates that play a crucial role in marine ecosystems by controlling benthic algal communities and whose natural populations are being affected by different biotic and abiotic factors. Triggering physiological processes promotes the activation of certain metabolic pathways, so oxidative status markers could be a suitable tool to asses maturation stage in which natural populations are. Antioxidant status of three species of Mediterranean Sea urchins, *A. lixula*, *P. lividus* and *S. granularis,* was evaluated in gonadal and digestive tissue. Superoxide dismutase (SOD), catalase (CAT), glutathione peroxidase (GPx), glutathione reductase (GR), glutathione s-transferase (GST), glucose 6-phosphate dehydrogenase (G6PDH), NAD(P)H: quinone oxidoreductase (NQO1) and lipid peroxidation were assayed. Significant differences were found among species, displaying in general higher antioxidant activity in *A. lixula* and *S. granularis* compared to *P. lividus*. A significant effect of sex was observed with females exhibiting a higher gonadosomatic index and higher levels of lipid peroxidation mainly in *A. lixula*. These results seem to be related to metabolic fluctuations associated with the gonadal maturation stage. Changes in digestive tissue were less evident, but some differences among species could be related to triggered digestive processes for replenishment of energy reserves in gonads. Oxidative status can be a useful complementary tool to evaluate gonadal condition in species of sea urchin from the same habitat. Integrative physiological and biochemical studies will contribute to the knowledge of invertebrate physiology.

## 1. Introduction

In marine ecosystems, commercial fishing represents major anthropogenic pressure leading to depleted shellfish populations. Additionally, unusual increases in sea temperature leading to a consequent water tropicalization have facilitated the expansion of invasive species, such as brown algae *Rugulopteryx okamurae* [1,2] reducing biodiversity by affecting Natura 2000 areas [3]. In parallel, thermal anomalies represent a major environmental stressor capable of triggering acute physiological disturbances in marine organisms, particularly in ectothermic invertebrates whose biological processes are tightly regulated by ambient temperature [4].

Sea urchins (Echinodermata) are key organisms of Mediterranean subtidal ecosystems, playing a central role in controlling benthic algal communities [5]. *Paracentrotus lividus* (Lamarck 1816) is one of the most exploited species of echinoderms in the Mediterranean Sea due to the traditional gastronomic demand of its gonads and whose populations are being affected up to 65% in southern Spain [5,6]. In Mediterranean seabeds *P. lividus* coexists with two other species, *Arbacia lixula* (Linnaeus 1758) and *Sphaerechinus granularis* (Lamarck 1816), the latter less commonly harvested but with the decline of *P. lividus* populations, it has become an alternative for local consumption. Due to the critical situation of sea urchin populations in the south of Spain, the authorities decreed the closure of the fishery for the common sea urchin *P. lividus* within this area [7]. To this sense, the General Fisheries Commission for the Mediterranean (GFCMs) of the Food and Agriculture Organization of the United Nations (FAO) highlights the need to deal with the decline in natural stocks and the relevance of evaluating and preserving reproductive potential of sea urchins [8].

Sea urchins have been widely used as biological markers to assess the effects of both classic and emerging environmental stressors and seawater alterations, such as pH, chemical pollutants or microplastics [9,10]. Due to the thermophilic nature of their larvae, biological processes can be activated by abiotic signals that may depend primarily on photoperiod and temperature [11].

In marine invertebrates, seasonal variations in metabolic rate are assumed to entail corresponding alterations in reactive oxygen species (ROS) formation [12]. Gonadal maturation is a seasonal physiological process that involves the mobilization of energy reserves for gametogenesis, entailing a metabolic activation that can trigger the production of reactive oxygen species (ROS) [13]. In sea urchins, a high antioxidant response has been demonstrated during the stages associated with the peak of gamete formation [10] which could also occur as a defense strategy during the growing phase of gametogenesis, to ensure good offspring quality [9]. Thus, the molecular mechanism of ROS in sea urchin gonads involves a delicate balance between essential signaling for maturation, environmental stress-induced damage, and robust antioxidant defense mechanisms [4]. On the other hand, sea urchin gonads act also as energy reserve tissue [14], establishing a clear dependence on digestive function and depending on external factors like food availability, interspecific competition or anthropogenic pollutants that can affect metabolic pathways and promote oxidative processes [15].

Unchecked ROS accumulation can impair metabolic enzyme activity, reduce ATP production, disrupt protein structure, and compromise cellular membrane integrity, ultimately promoting apoptotic events [16]. To protect tissues from the harmful effects of ROS and mitigate oxidative damage, organisms have a complex antioxidant defense system. Enzymatic defenses include superoxide dismutase (SOD) and catalase (CAT), with the ability to remove superoxide anion and hydrogen peroxide, respectively. Glutathione peroxidase (GPX) provides protection by detoxifying hydrogen peroxide and organic peroxides. Glucose-6-phosphate dehydrogenase (G6PDH) is a crucial enzyme providing NADPH, essential for regeneration of glutathione, by glutathione reductase (GR) activity, which converts oxidized glutathione (GSSG) into its reduced form (GSH), neutralizing the damaging effects of free radicals. The enzymes NQO1 (previously defined as DT-diaphorase) and GST have both been reported to increase their activity in aquatic organisms under the effect of pollutants [17,18]. In this context, the assessment of oxidative status using biomarkers of damage and defense (e.g., MDA, total antioxidant capacity, and antioxidant enzymes) has been proposed as a key indicator of physiological status in marine invertebrates, as it integrates the response to multiple environmental stressors and links it to reproductive performance parameters [19].

Since physiological processes of reproduction promote higher energy costs associated with a metabolic activation, the aim of this study is to conduct a comparative study that correlates antioxidant status markers and gonadal maturation among three species of sea urchin from the Mediterranean Sea coast under the same environmental conditions. Integrative physiological and biochemical studies will contribute to the knowledge of invertebrate physiology and could be useful to predict future trends during the reproductive period and develop strategies aimed to promote conservation strategies of natural populations and sustainable fishery management plans.

## 2. Materials and Methods

### 2.1. Specimens and Sample Collection

**Figure 1:**
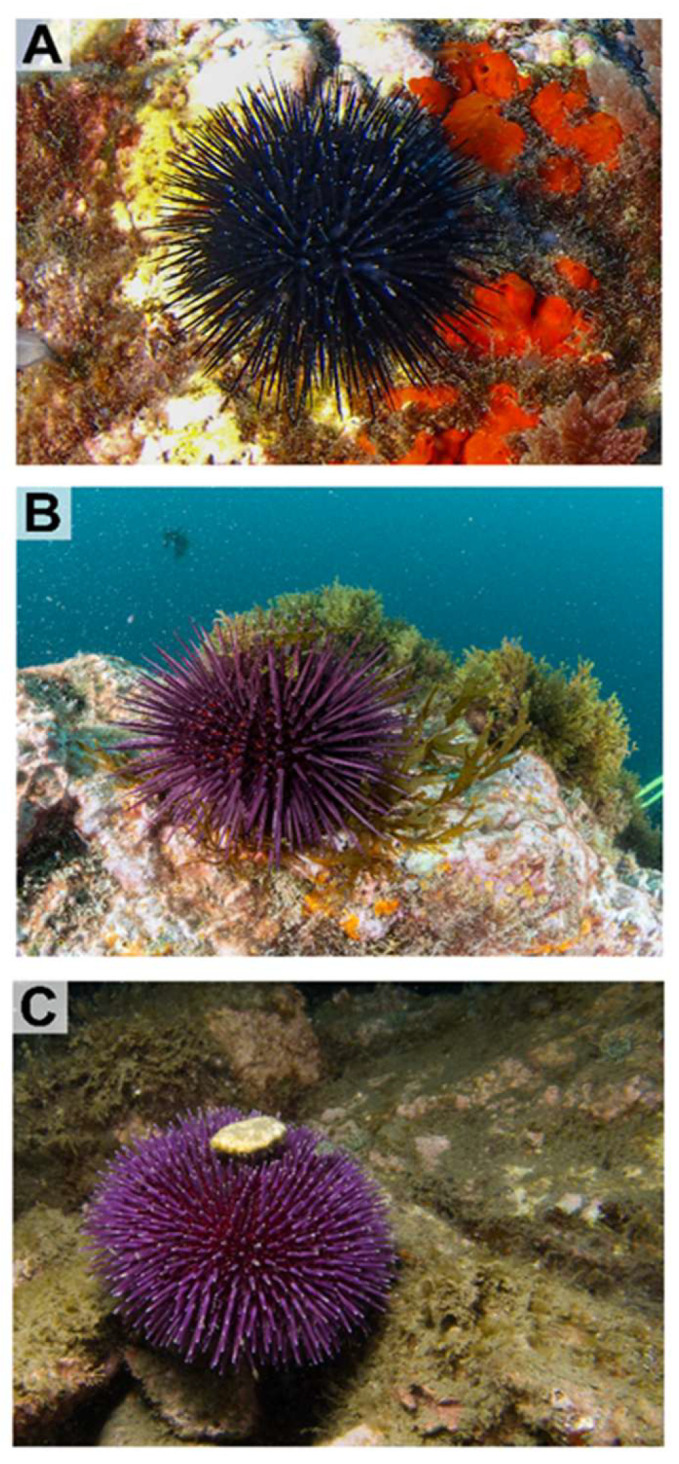
Regular sea urchin species of Mediterranean coasts collected in this study: (**A**) *Arbacia lixula*; (**B**) *Paracentrotus lividus*; (**C**) *Sphaerechinus granularis*.

The following three Mediterranean species of sea urchin present in the rocky infralittoral zone of the Calahonda (Granada, Spain; 36°42′09.5″ N, 3°24′43.1″ W) were sampled: *Arbacia lixula* (1–3 m depth), *Sphaerechinus granularis* (1–7 m depth), and *Paracentrotus lividus* (1–3 m depth) (Figure 1). The sampling point is significant due to its biological diversity and hosts one of the richest and most diverse underwater fauna and flora heritages in Europe.

**Figure 2:**
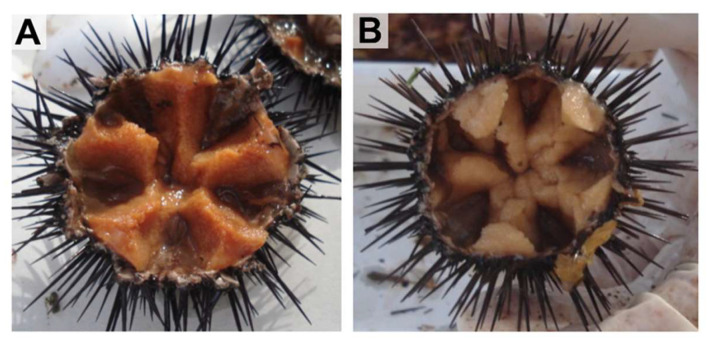
Sampled gonads of *Paracentrotus lividus* in (**A**) female specimen and (**B**) male specimen.

The samples were collected manually from the natural environment in May by scuba diving. Water condition was 17 °C temperature, 37 ‰ salinity, and dissolved oxygen levels above saturation. A total of six individuals of each species (3 males and 3 females) were selected, adjusting the minimum number of collected specimens allowed by responsible authorities for carrying out this experimental assay in nature populations. Animals were measured and weighed, and ex was determined in wet samples of gonads (Figure 2) after they had been crushed and processed by a smear on a slide, checking for the presence of sperm or eggs under optical microscopy (Leica DM 750). Gonadal and digestive tissues were dissected and weighed to obtain the gonadosomatic index (GSI) calculated as (gonad wet weight (g) × 100)/total wet weight (g) and the digestive somatic index (DSI) as (digestive wet weight (g) × 100)/total wet weight (g) according to Martínez-Pita et al. [20]. Thereafter gonadal and digestive tissues were immediately frozen in liquid nitrogen and stored at −80 °C until processing.

### 2.2. Determination of Oxidative Status

Samples of gonads and extragonadal tissue (digestive) were homogenized (Heidolph Instruments, Schwabach, Germany) in 100 mM Tris, 0.1 m EDTA and 0.1% Triton buffer (pH 7.8), at a 1:4 (*w*/*v*) ratio. Homogenates were centrifuged at 30,000 g for 30 min at 4 °C (Sigma 3K30, St. Louis, MO, USA), and the supernatant was collected and distributed in aliquots kept at −80 °C.

Superoxide dismutase (SOD; EC 1.15.1.1) activity was analyzed using the McCord and Fridovich [21] method, which is based on an indirect measurement of activity in relation to the degree of inhibition of a control reaction based on cytochrome c reduction. Catalase (CAT; EC 1.11.1.6) activity was determined using the method originally described by Aebi [22], based on the decrease in absorbance resulting from the degradation of H_2_O_2_. Glutathione peroxidase (GPx; EC 1.11.1.9) activity was measured using the Flohé and Günzler [23] method, which is based on an indirect measurement of NADPH oxidation, obtained by coupling with a standard glutathione reductase (GR; EC 1.8.1.7) reaction. GR activity was determined using the Carlberg and Mannervik [24] method, which is based on the decrease in absorbance caused by NADPH oxidation. Glucose 6-phosphate dehydrogenase (G6PDH; EC 1.1.1.49) activity was assayed measuring the change in absorbance due to NADPH production following a modified method of Lohr and Waller [25]. The glutathione S-transferase (GST; EC 2.5.1.18) activity of the samples was determined following the method of Frasco and Guilhermino [26] measuring the increase in absorbance due to the formation of a conjugate between glutathione and 2,4-dinitrochlorobenzene. NAD(P)H: quinone oxidoreductase 1 (NQO1; EC 1.6.5.2.) activity was determined using a modified method of Lemaire et al. [27], based on measurement of the decrease in absorbance caused by reduction of 2,6-dichlorophenol indophenol.

For all the enzymes, except SOD, one unit of activity was defined as the amount of enzyme required to transform one μmol of substrate per minute under the measurement conditions. For SOD, one unit of activity was defined as the amount of enzyme required to generate a 50% inhibition in the reduction in cytochrome c.

The enzymatic activities were expressed as specific activity, for which the soluble protein content in the samples was quantified according to Bradford [28]. Finally, lipid peroxidation was measured based on the content of thiobarbituric acid reactive substances (TBARSs) according to a modified method of Buege and Aust [29]. For the determination of TBARS, malondialdehyde (MDA) was used as a standard.

All measurements were performed with a PowerWave microplate spectrophotometer (Bio-Tek Instrument, Inc., Winooski, VT, USA).

### 2.3. Statistical Analysis

A two-way ANOVA was used to detect differences in means associated with sea urchin species and sex. ANOVA assumptions were checked via Shapiro–Wilk test and Levene test. When homoscedasticity was not met, a logarithmic transformation was applied to the response variable. Significant main effects were followed up by Tukey’s test as *post hoc* test when appropriate. Benjamini–Hochberg correction was used to adjust *p*-values and prevent error inflation due to multiple testing. Statistical significance level was set at α = 0.05

Principal Component Analysis (PCA) was performed on gonadal variables as a descriptive technique to examine correlation between measured variables and tendencies at the multivariate level. Digestive tissue variables were excluded due to low adequacy for PCA according to Kaiser–Meyer–Olkin test. Barlett’s test of sphericity was used to test the assumption that the correlation matrix was different from an identity matrix.

## 3. Results

### 3.1. Gonadosomatic and Digestive Somatic Index

**Figure 3:**
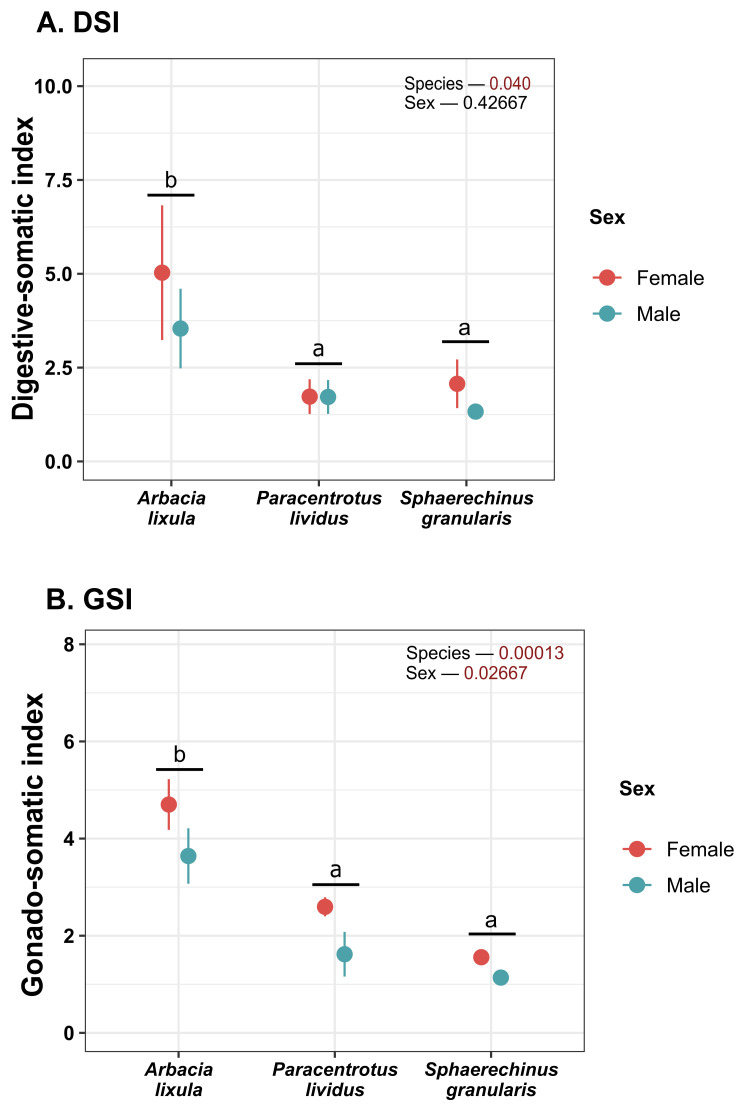
Plot of (**A**) digestive-somatic index (DSI) and (**B**) gonadosomatic index (GSI). Values correspond to mean ± SEM. a, b: differences between species regardless of sex.

Both gonadosomatic index (GSI) and digestive-somatic index (DSI) were higher in *Arbacia lixula* than in other species (Figure 3). Female sea urchins also displayed higher GSI than males.

### 3.2. Gonadal Tissue Oxidative Status

**Figure 4:**
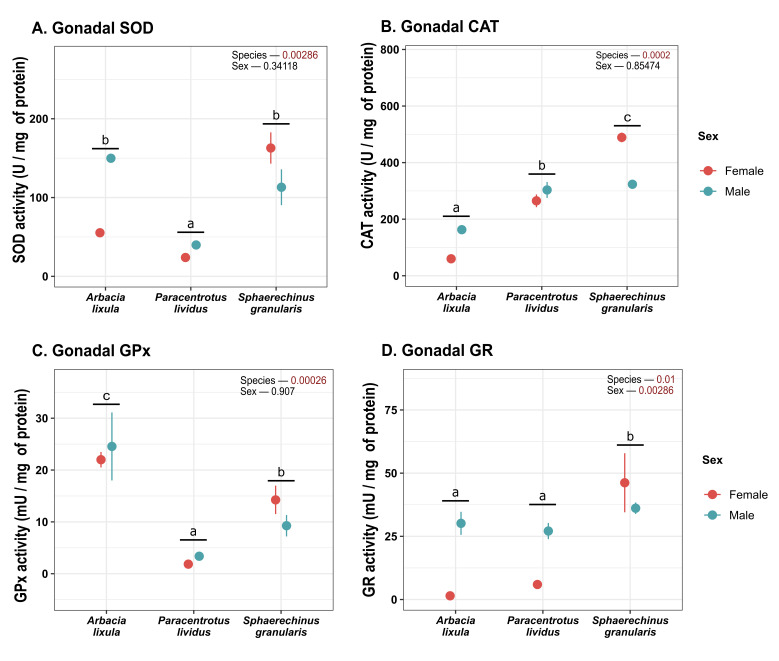
Plot of (**A**) CAT; (**B**) SOD; (**C**) GPx and (**D**) GR activity in gonads. Values correspond to mean ± SEM. a, b, c: differences between species regardless of sex. CAT: catalase, SOD: superoxide dismutase, GPx: glutathione peroxidase, GR: glutathione reductase.

*S. granularis* and *A. lixula* had higher SOD activity (Figure 4A) than *P. lividus.* There were no overall differences between sexes. CAT levels in gonadal tissue varied significantly across species (Figure 4B). *A. lixula* displayed the lowest activity of the three species, and *Sphaerechinus granularis* featured the highest. GPx activity was significantly different across species (Figure 4C). *A. lixula* displayed the highest activity, while *P. lividus* featured the lowest. Regarding GR (Figure 4D), male sea urchins had higher activity than females and *S. granularis* featured higher activity than other species.

**Figure 5:**
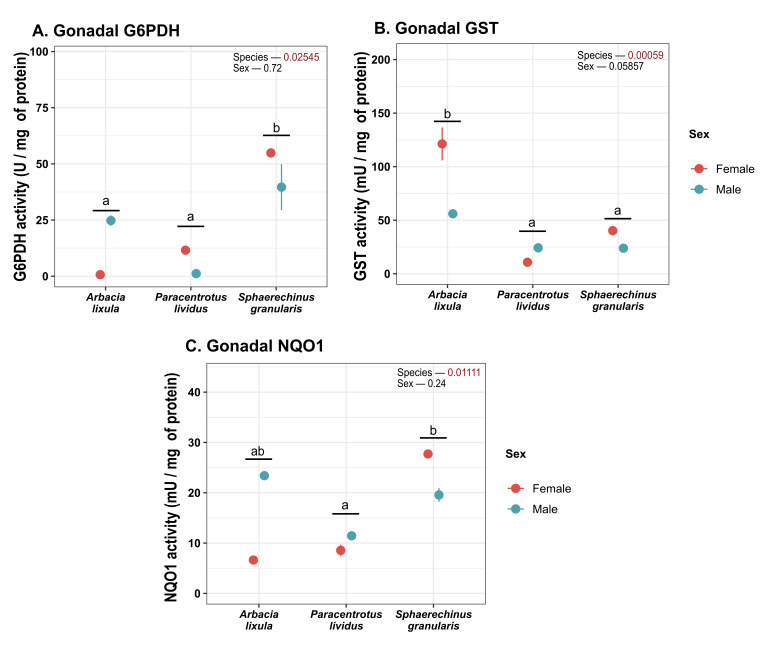
Plot of (**A**) G6PDH; (**B**) GST and (**C**) NQO1 activity in gonads. Values correspond to mean ± SEM. a, b: differences between species regardless of sex. G6PDH: glucose 6-phosphate dehydrogenase), GST: glutathione S-transferase), NQO1: NAD(P)H: quinone oxidoreductase 1.

G6PDH activity was higher in *S. granularis* sea urchins (Figure 5A), while GST activity was found to be higher in *A. lixula* (Figure 5B). NQO1 activity was higher in *S. granularis* than in *P. lividus* (Figure 5C).

**Figure 6:**
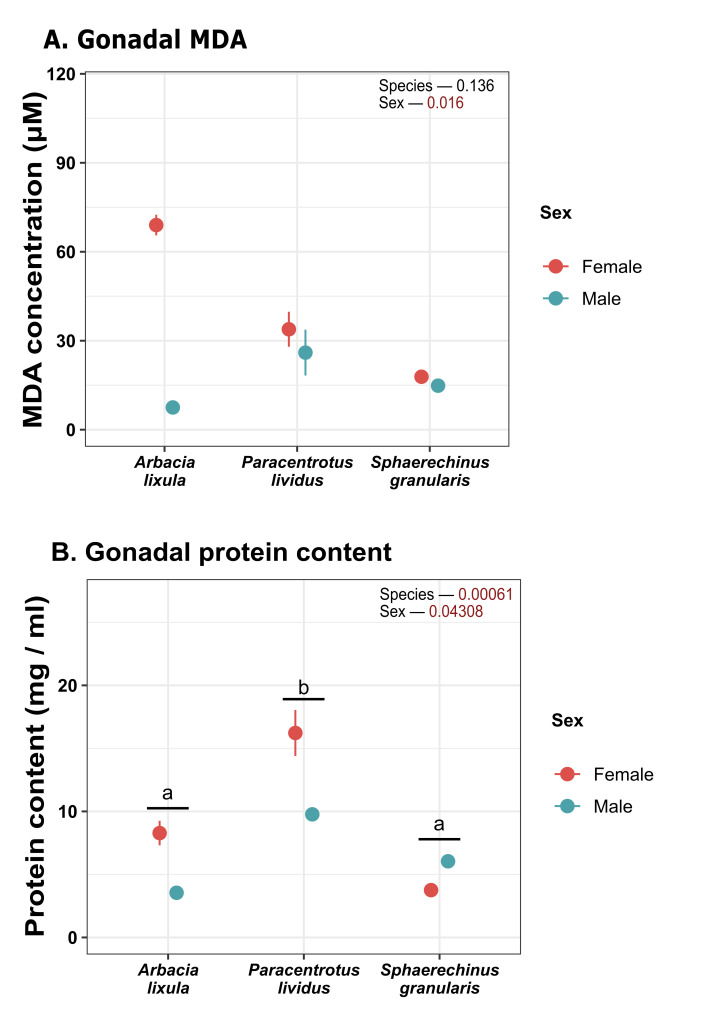
Plot of (**A**) lipid peroxidation measured as MDA; (**B**) protein concentration in gonads. Values correspond to mean ± SEM. a, b: differences between species regardless of sex. MDA: malondialdehyde.

Regarding lipid peroxidation (Figure 6A), females had overall higher MDA content than males in gonadal tissue (Figure 6A). This difference was particularly pronounced in *A. lixula*, but there were no overall statistical differences between species. Soluble protein levels in gonadal tissue (Figure 6B) were generally higher in females, and *P. lividus* had the highest protein content for both sexes (16.22 mg/mL in females and 9.77 mg/mL in males).

### 3.3. Digestive Tissue

**Figure 7:**
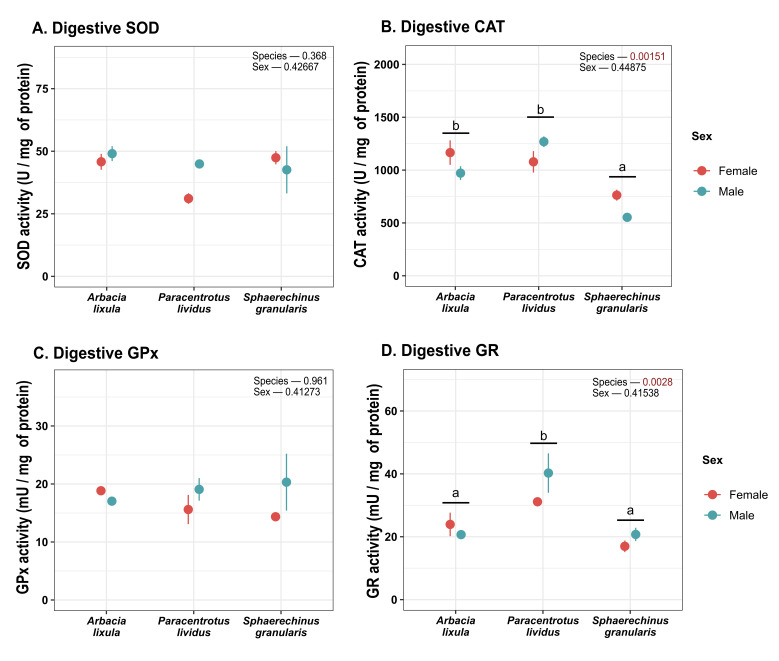
Plot of (**A**) CAT (A); (**B**) SOD, (**C**) GPx and (**D**) GR activity in digestive tissues. Values correspond to mean ± SEM. a, b: differences between species regardless of sex. CAT: catalase, SOD: superoxide dismutase, GPx: glutathione peroxidase, GR: glutathione reductase.

**Figure 8:**
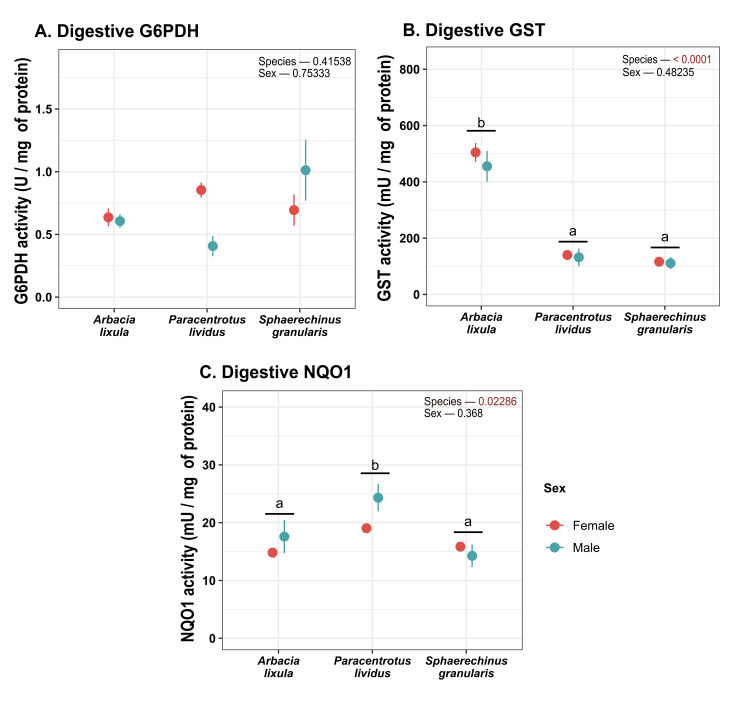
Plot of (**A**) G6PDH; (**B**) GST and (**C**) NQO1 activity in digestive tissues. Values correspond to mean ± SEM. a, b: differences between species regardless of sex. G6PDH: glucose 6-phosphate dehydrogenase), GST: glutathione S-transferase, NQO1: NAD(P)H: quinone oxidoreductase 1.

Catalase activity in digestive tissues (Figure 7) was found to be lower for *S. granularis* sea urchins. In contrast, neither SOD, GPx nor G6PDH activity differed between sea urchin species or sexes in digestive tissue. GR activity was significantly higher for *P. lividus* (Figure 7 and Figure 8). GST activity (Figure 8) was higher in *A. lixula* than other sea urchin species. *P. lividus* sea urchins were also found to display higher NQO1 activity (Figure 8) than other species.

**Figure 9:**
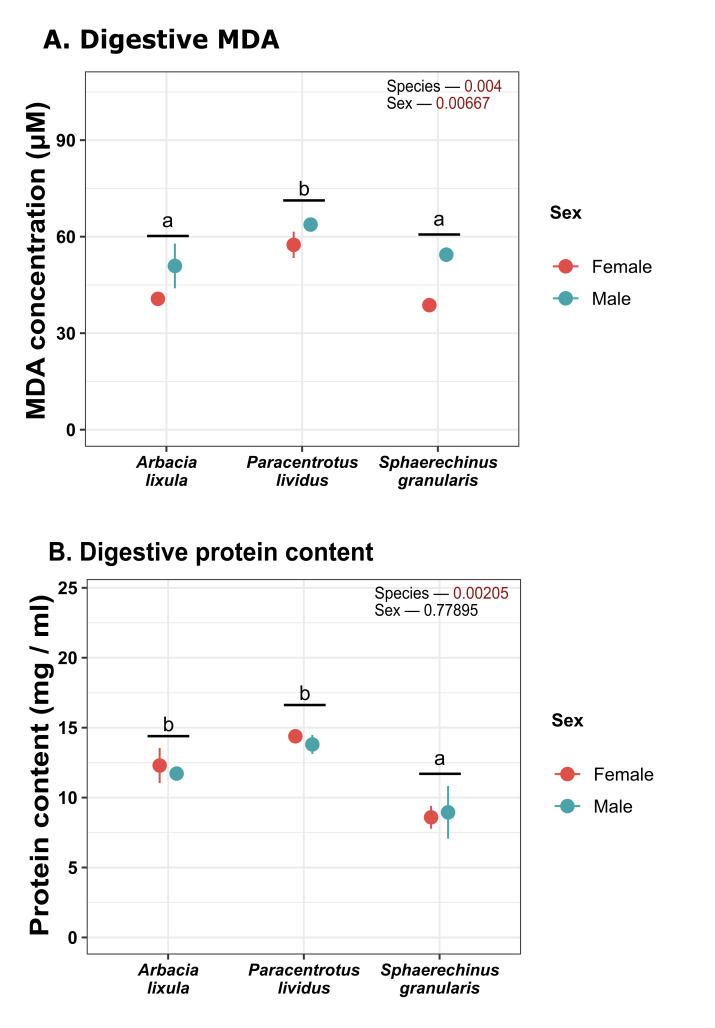
Plot of (**A**) lipid peroxidation measured as MDA; (**B**) protein concentration in digestive tissues. Values correspond to mean ± SEM. a, b: differences between species regardless of sex. MDA: malondialdehyde.

Regarding lipid peroxidation (Figure 9), male sea urchins overall featured higher MDA concentration and there were also differences across species, as *P. lividus* displayed higher peroxidation levels. Soluble protein (Figure 9) content was found to be lower in *S. granularis*.

### 3.4. Principal Component Analysis

**Figure 10:**
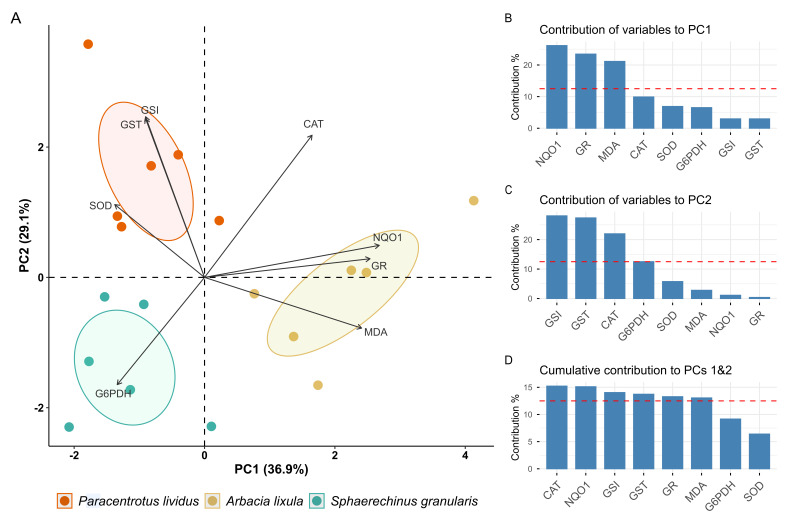
Principal Component Analysis of gonadal variables: (**A**) PCA biplot with samples grouped based on species, with confident ellipses build on a confidence level of 0.95; (**B**) percentage contribution of the variables to PC1; (**C**) percentage contribution of the variables to PC2; (**D**) cumulative contribution of the variables to both PC1 and PC2. Red dashed lines represent expected average contribution.

Principal Component Analysis (PCA) was performed on measured gonadal parameters to explore correlation between variables, excluding GPx based on low KMO score that would impact the analysis. Figure 10A illustrates the biplot based on the first two principal components (PC1 and PC2). Observations were grouped based on species, and variable vectors were overlaid on top of the biplot (Figure 10A). PC1 explained 36.9% of initial variance, while PC2 accounted for 29.1% of it. Together, they cumulatively explained 66% of data variance. The histogram in Figure 10B shows variables contribution to PC1, which was mainly spread among NQO1, GR and MDA. All of these variables showed a higher contribution than expected by chance (dashed line) and contributed positively to PC1, indicating positive correlation among them. On the other hand, GSI, GST, CAT and G6PDH were the major contributors to PC2 (Figure 10C), and only G6PDH contributed negatively, indicating negative correlation with GST, CAT and GSI. Overall, CAT, NQO1, GST, GSI, GR and MDA showed high cumulative influence on both PC1 and PC2 (Figure 10D).

## 4. Discussion

In recent years, physical and chemical changes in the environment, invasive species, and anthropogenic activity have had a negative impact on marine ecosystems and, consequently, on invertebrate species such as sea urchins, which play a crucial role in regulating algal populations due to their position as primary consumers in the food web [30,31].

In the development of restorative actions for aquatic organisms, the assessment of physiological response under particular environmental conditions could be a suitable tool for establishing optimal culture conditions, as was previously evaluated in other invertebrates such as sea anemone [19].

Most echinoderm species show remarkable natural fluctuations which may be related to the regulation of their reproductive processes by external factors [32]. Environmental conditions play a critical role in the maturation, reproduction, and development of sea urchins, with water temperature, food availability, and pH (acidification) being the most influential factors [33]. These factors determine the timing of spawning, the rate of gonadal maturation, and larval quality. On the other hand, several studies have stated that sea urchin response to environmental changes can manifest through changes in oxidative status parameters associated with an adaptive response [9,10].

During the reproductive cycle of sea urchins, the gonads undergo macroscopic changes in size and histological organization and experience a series of established stages after spawning as follows: (i) vitellogenesis and accumulation of energy reserves in specialized cells called nutritive phagocytes; (ii) gamete formation and descent of nutritive phagocytes; (iii) gamete accumulation (pre-spawning) and release (spawning); and finally (iv) gonadal reconstitution based on the formation of nutritive covers. These four stages, which are easily recognizable in females, are reduced to the following three stages in males: (I) accumulation of reserves, (II) formation of gametes, and (III) accumulation of gametes and release of gametes [34,35]. The gonadosomatic index (GSI) of mature sea urchins is a critical indicator of reproductive development [36].

In this study, the differences displayed in GSI could initially be attributed to the different degrees of maturation between species at the time of collection. However, similar behavior displayed in the digestive somatic index (DSI) among species leads us to believe that these differences could instead be attributable to the different relative body diameter (thus weight) rather than significant changes in gonadal or digestive tissue weight. Therefore, although the GSI measurement may be a criterion of interest to assess the state of gonadal maturation [30], when it comes to establishing a comparison between species at the same point in time, it does not appear to be as consistent. In this regard, the analysis of biochemical markers may be a complementary strategy for understanding the state of maturation of different species from a single environment at a single time point.

Sea urchin gonads, besides having a reproductive function, are also capable of acting as reserve organs, with both processes being closely related [14]. Thus, the analysis of digestive tissue can provide information of interest regarding adaptations aimed at meeting greater or lesser energy demands during maturation stages. Reserve cells in gonads, known as nutritive phagocytes, accumulate proteins, lipids and carbohydrates necessary for gamete formation processes and for their metabolic mobilization as an energy source in case of need due to low food availability. These cells can also reabsorb gametes not expelled after spawning as a source of energy [30].

During the stages of gonadal maturation, the activation of gametogenesis involves energy expenditure from gonadal reserves, which promotes the activation of certain metabolic pathways, increasing oxidative processes [37]. This can promote the formation of reactive oxygen species (ROS) and generate a protective response in organisms based on molecular and enzymatic antioxidant defenses [38,39].

Perez et al. [40] demonstrated a positive correlation between gonadal maturation and lipid soluble antioxidant concentration in marine invertebrates, such as the starfish *Anasterias antarctica*, as a strategy for oxidative damage prevention and gamete protection through allocation of antioxidants to mature gonads.

This evaluation of antioxidant enzymes in three species of sea urchin present on the Mediterranean coast—*A. lixula*, *P. lividus*, and *S. granularis*—displayed differences that could be attributed to the stage of gonadal maturation each of them was in. It is known that during the peak of gametogenesis, sea urchins maintain higher antioxidant levels [10]. One notable thing was that the black sea urchin, *A. lixula*, showed significantly higher values of the enzymatic antioxidant activity, glutathione peroxidase (GPx) and glutathione transferase (GST). This could be attributed to the fact that this species was in a gonadal stage in which gamete formation was relevant. Interestingly, for both enzymes activity is necessary reduced glutathione (GSH), which is involved in phase II metabolism that plays a crucial role in intracellular protection against reactive oxygen species [41]. According to Lubos et al. [42] the intracellular content of glutathione depends on environmental factors and is maintained in equilibrium between its utilization and its synthesis, an aspect that could be in agreement with environmental effect on maturation processes. On the other hand, GPx shares with catalase (CAT) the function of removing inorganic peroxides. The low activity of the latter could indicate that GPx would have primarily assumed this role triggered by a physiological stage. This would be consistent with the fact, suggesting that the glutathione redox cycle is an important protective mechanism against mild oxidative stress, being catalase more crucial protecting cells against chronic oxidative stress [43].

On the other hand, it is important to highlight the role of GPx removing organic peroxides, like lipoperoxides. Lipids, such as fatty acids (FAs) and polyunsaturated fatty acids (PUFAs), are present in sea urchin gonads and are essential for their physiological processes [34,44]. Several studies have shown an increase in lipid during the stages of gonadal maturation, which then undergoes a clear decline in the final stages and after spawning [45,46]. In this study, females showed higher MDA values in gonadal tissue. The accumulation of lipids in the female gonads of sea urchins is a sex-specific process directly linked to the reproductive cycle, in which females store significantly higher levels of total lipids and PUFAs compared to males. These lipids, which constitute essential energy sources for oogenesis (egg formation) and larval development, reach their peak level before spawning and declining and decreasing and becoming equal in both sexes after spawning [47]. To this respect, Alonso-Alvarez et al. [48] suggested that reproduction might generate oxidative stress, increasing lipid radical content in mature gonads compared to immature gonads and that, in the long term, could affect the reproductive potential of the gonad. Pérez et al. [37] reported an increase in the concentration of TBARS (thiobarbituric acid reactive substances) and lipid radicals during gametogenesis, associated with a decrease in the levels of fat-soluble antioxidants (tocopherol, carotene and equinenone). The trend to higher MDA values in females and particularly in *A. lixula* (Figure 6) could be consistent with a greater accumulation of PUFAs, fatty acids with a high propensity for oxidation, justifying in this species an increased antioxidant requirement in agreement with higher values of GPx.

The results suggest that *A. lixula*, at the time of capture in May, was in a period of gonadal maturation and gamete formation linked to high metabolic activity promoting ROS generation. This agrees with the fact that the gonad growth period of *A. lixula* on Mediterranean coasts has been reported to occur from winter to spring, with most individuals maturing during May–June and spawning from spring to late summer/early autumn, depending on seasonal temperatures [49]. Also, GSI values in *A. lixula* in this study are within the same range (around 4–5%) reported by these authors at the same season time [49,50].

The evaluation of antioxidant activity in the gonads of the common sea urchin, *P. lividus*, curiously revealed a trend opposite to that observed in *A. lixula* for the same enzymes. This response had already been observed in marine invertebrates such as the mussel *Mytilus galloprovincialis*, where a progressive decrease in antioxidant activity was observed after egg laying despite the availability of food and higher temperatures [51]. This could be consistent with the fact that *P. lividus* could be in a post-spawning stage at the time of sampling, supported by Uz et al. [52], reporting that in *P. lividus* from the northeast coast of Spain had already spawned during the months of May–June. At this stage, the gametogenic processes linked to the activation of ROS-producing metabolic pathways would not be a priority, thus justifying a decrease in endogenous antioxidant defenses. In addition, after gonadal spawning, there is a depletion in lipid energy reserves [47], which would justify the low MDA concentration observed in *P. lividus* and the relative higher protein concentration in gonads of these animals.

The gonadosomatic index values obtained in *P. lividus* were consistent with those obtained by Raposo et al. [53] (1.5–3%) in individuals of similar size (diameter of 4–5 cm), under the same seasonality (May–June) and temperature (17 °C) in a post-spawning period, being low when compared to other indices that range from 8 to 12% in periods of maturity prior to spawning. This could be indicative that in the animals, at the time they were collected, spawning had probably already taken place, with the gonads presenting a low number of residual gametes and being in an initial stage of energy reserve accumulation.

The violet or purple sea urchin, *Sphaerechinus granularis*, is an edible species of increasing commercial importance that coexists in Mediterranean waters with *P. lividus* and *A. lixula*, although it is not as abundant as the latter two. The general increased activity in most of the enzymes evaluated (SOD, CAT, GPx GR, G6PDH, and NQO1), which tended to be more pronounced in females, could be consistent with the spawning period for this species, which begins in June and can extend until November, with a peak on the southern coast of the Iberian Peninsula in the months of June–August [20]. This could explain why the animals were still in the middle of gametogenesis, with the consequent tendency to generate ROS [37], supporting the high antioxidant activity in most of the enzymes evaluated. This is consistent with reports by Perez et al. [39] in sea urchin, showing an increased lipid radical/a-TH index during preceding spawning stage that could be associated with a higher metabolism in the lipid phase of the gonads. In this study, MDA levels were low, which could be indicative that the antioxidant response was effective in preventing lipid oxidation.

According to GSI variations associated with gonadal maturation, Martinez-Pita et al. [20] reported values ranging from 3 to 5%. for *S. granularis* in southern Spain during a similar seasonal period of this study. The lower GI values displayed for animals (less than 2%), given the period and water temperature when animals were sampled, it does not appear to be attributable to gamete release stage. However, these GI values agree with Vafidis et al. [54] in specimens from the Aegean coast under the same seasonality and which were in previous stages with gamete formation and maturation. This seems more likely and would justify the increase in gonadal metabolic activity and, therefore, the antioxidant enzyme response. According to the same authors, the trend to more pronounced antioxidant response in females could be due to the advance in maturation processes compared to males. In addition, female sea urchins generally have a higher gonadosomatic index (GSI), often with a higher lipid and carotenoid content, as they invest more energy in egg production [55]. This would support the fact that in this study, females, regardless of species, showed significantly higher GSI than males.

Another factor that could contribute to the low gonadal growth of *S. granularis* is the reduced availability of food for this species [20,56,57]. Itshould be noted that in Mediterranean waters, the predominant species are *A. lixula* and *P. lividus*, whose diet is based on encrusting coralline algae and marine phanerogams and algae from rocky areas, respectively [6,58]. *S. granularis* inhabits deeper areas and prefers feeding on decaying plant material and epiphytic algae. Low food availability may influence gonad size in *S. granularis*. These results suggest investment in gonadal growth under high hydrodynamics and probably less favorable food supply [54]. In this sense, food supply would be key in promoting the physiological activity of the digestive tissue, to ensure the replenishment of energy reserves, in which the gonad, as mentioned above, would play a prominent role as reserve tissue.

The results of the assessment of oxidative status in intestinal tissue were not as relevant as those observed in the gonads, nor did they show any sex-related effect. This was to be expected, as metabolic changes in digestive tissue are less pronounced than in gonads. However, certain activation of oxidative processes (CAT, GR and NQO1) linked to a trend to higher lipid hydroperoxides in *P. lividus* could be consistent with an activation of digestive activity. The fact that this species, as previously hypothesized, could be in a post-spawning period would have justified a metabolic activation for gonadal regeneration and energy reserve replenishment, triggering oxidative processes and antioxidant requirements [59]. On the other hand, during stages prior to egg laying and gonadal maturation, the intestinal function for the regeneration of energy reserves is not yet a priority [34,35,60]. This would be consistent with the lowest levels observed in the digestive tract of the other two species (*A. lixula* and *S. granularis*) in terms of lower antioxidant activity and lipid peroxide concentration.

GST activity is an enzyme mainly involved in detoxification processes through the formation of soluble conjugates [61] and previously it has been reported to be a good indicator of environmental stress in sea urchin [62]. *A. lixula* usually colonizes rocky areas at shallower depths and in this study the sampling point was a dock area where there are hydrocarbons from fuels and agricultural pesticides, favoring the accumulation of xenobiotic agents associated with anthropogenic activity. The marked increase displayed in the intestinal tissue of *A. lixula*, regardless of sex, is consistent with previously observed for the gonads. This response could be consistent with the reported high capacity of this species to bioaccumulate contaminants [62]. The fact that both tissues (gonadal and digestive) had a similar behavior could reflect the greater sensitivity of this species, explaining the oxidation of gonadal lipids manifested by higher levels of MDA [10].

Principal Component Analysis (PCA) performed on measured gonadal parameters showed that major contributors to PC1 (NQO1 activity, GR activity, and malondialdehyde concentration) are important drivers of the differences between the sampled sea urchins. Similarly, variables such as GST activity, CAT activity and gonadosomatic index (GSI), which were the major contributors to PC2, also are highlighted. Some antioxidant enzyme activities showed positive correlation, such as GR and NQO1, but these were also correlated with malondialdehyde (MDA), indicating that samples that had higher MDA levels also had higher activities of these two enzymes. SOD and GST activity, however, were negatively correlated with lipid peroxidation marker MDA, as well as GSI. There also appears to be some degree of negative correlation between CAT and G6PDH activity levels. *P. lividus* observations were defined by their higher GST activity and GSI and low MDA content. *S. granularis* sea urchins featured high G6PDH but low antioxidant activity and lipid peroxidation. Finally, *A. lixula* was the species with the higher spread in PC1, which suggests a highly variable oxidative state between males and females. They were characterized by higher levels of MDA, NQO1 activity, and GR activity compared to other species.

It is important to note that this study has put forward hypotheses that will need to be further supported in subsequent studies, given certain limitations such as the small number of specimens used in the evaluated parameters. Current regulation regarding sea urchin capture has been a limiting factor, and this, combined with the lack of phenotypic differences between sexes, has meant that specimens were collected only until a statistically minimum number was reached, ensuring a balanced sample size for each sex within each species.

On the other hand, despite the aim of this study being providing a comparative overview of the physiology of gonadal maturation in three species coexisting in the same location and at the same point in time, this objective must be further explored in more extensive studies including a seasonal analysis for each of the species studied, and supplemented with a histological evaluation of the gonads to support gonadal maturation stages described.

## 5. Conclusions

In short, taking a general look at the results obtained of the three species studied, *A. lixula* and *S. granularis* appear to show at the time of collection an antioxidant behavior consistent with a stage of gonadal maturation prior to spawning, in which metabolic processes would be activated by mobilization of energy reserves for gametogenesis. In contrast, *P. lividus* could be in a period of replenishment of energy reserves after spawning. The analysis of oxidative status markers could be a complementary tool for the evaluation of gonadal maturation status in sea urchins. The different behavior in the three species collected from a single environment at a single time point could be related to an endogenous component associated with their biology modulating gonadal processes.

## Data Availability

The raw data supporting the conclusions of this article will be made available by the authors on request.

## Abbreviations

The following abbreviations are used in this manuscript:

ROS	Reactive oxygen species
SOD	Superoxide dismutase
CAT	Catalase
GPx	Glutathione peroxidase
GR	Glutathione reductase
GST	Glutathione s-transferase
G6PDH	Glucose 6-phosphate dehydrogenase
NQO1	NAD(P)H: quinone oxidoreductase
MDA	Malondialdehyde
TBARS	Thiobarbituric acid reactive substances
GSI	Gonadosomatic index
DSI	Digestive somatic index
PCA	Principal Component Analysis
